# Indolent lymphoma with composite histology and simultaneous transformation at initial diagnosis exhibit clinical features similar to *de novo* diffuse large B-cell lymphoma

**DOI:** 10.18632/oncotarget.24701

**Published:** 2018-04-13

**Authors:** Hanno Witte, Harald Biersack, Svenja Kopelke, Dirk Rades, Hartmut Merz, Veronica Bernard, Hendrik Lehnert, Niklas Gebauer

**Affiliations:** ^1^ Department of Hematology and Oncology, University Hospital of Schleswig-Holstein, Campus Luebeck, Luebeck, Germany; ^2^ Department of Radiation Oncology, University Hospital of Schleswig-Holstein, Campus Luebeck, Luebeck, Germany; ^3^ Hämatopathologie Lübeck, Reference Center for Lymph Node Pathology and Hematopathology, Lübeck, Germany

**Keywords:** transformed indolent lymphoma, composite histology, de novo diffuse large B-cell Lymphoma, cell-of-origin, prognosis

## Abstract

While various studies characterized clinical and prognostic properties of *de novo* diffuse large B-Cell lymphoma (DLBCL) and transformed indolent lymphomas, the clinicopathological features of indolent lymphoma and simultaneous secondary transformation upon initial diagnosis (ssDLBCL) are insufficiently established.

Between 2010 and 2017, 247 consecutive patients admitted to our institution and treated for DLBCL were investigated for composite histology of ssDLBCL-type. Upon systematical histopathological evaluation composite histology was identified in 22/247 cases (8.9%).

The predominant histology of the underlying indolent lymphoma was follicular lymphoma of variable grading (I-IIIA; 81.8%) whereas marginal zone lymphoma represented a minor sub group (18.2%). Clinicopathological investigation revealed a high degree of concordance between ssDLBCL and *de novo* DLBCL upon initial diagnosis and clinical courses were shown to be strikingly similar. The predominant fraction of ssDLBCL were germinal center derived lymphomas (GCB-type) with a trend towards a superior outcome compared with non-GCB-type ssDLBCL. Additionally, we demonstrate a significant adverse prognostic impact of an underlying indolent lymphoma component other than follicular-type lymphoma (e.g. marginal zone lymphoma). Moreover, the frequency of double-hit (DHL) or double-expressor lymphomas (DEL) appears to be low.

Our findings provide substantial insight into the behavior of ssDLBCL, highlight the ramifications of the concurrent high-grade fraction within indolent lymphomas and underline therapeutic efficacy of R-CHOP type immunochemotherapy in the majority of ssDLBCL patients.

## INTRODUCTION

The development of aggressive non-Hodgkin's lymphoma (typically DLBCL) in patients with underlying indolent lymphoma is commonly referred to as transformation [1, 2]. Diffuse large B-cell lymphoma (DLBCL) is the most common type of non-Hodgkin's lymphoma (NHL), and its incidence is closely correlated with increasing age [3]. In most studies this entity is subdivided into the categories of *de novo* DLBCL and transformed high-grade lymphomas with a preceding history of indolent (most commonly follicular and marginal zone derived) lymphoma. Studies predating the introduction of immunochemotherapy found follicular lymphoma that transformed to DLBCL (tFL) as well as other secondary high-grade B-cell lymphomas to portend a dismal prognosis with a median overall survival (OS) of approximately two years [4, 5]. Common indicators of transformation in primarily indolent NHL are raised LDH level, alternative biochemical surrogate markers for elevated cellular turnover (e.g. hypercalcemia, hyperuricaemia), rapid nodal/extranodal growth, swiftly diminishing performance status or increasing B symptoms as well as newly emerging sites of extranodal manifestation [6–9]. Clinical and prognostic data on indolent lymphoma with composite histology and simultaneous secondary transformation at initial diagnosis (ssDLBCL) or “transformed lymphoma at diagnosis” in the era of immunochemotherapy remain sparse as most prior studies on both indolent lymphoma as well as DLBCL neglected these patients. Despite the deficiency of prospective data in this group of patients, immunochemotherapy first-line treatment of aggressive lymphoma-type (e.g. Rituximab, Cyclofosfamide, Doxorubicine, Vincristin & Prednisolone (R-CHOP)) occasionally followed by high-dose chemotherapy and autologous stem cell transplantation (ASCT) has emerged as the therapeutic strategy of choice [10–12]. Only very recently a large-scale case study by Magnano *et al.* was published concluding that the outcome of ssDLBCL patients may not be worse than that of *de novo* DLBCL questioning the need for widely applied intensification with ASCT following induction by immunochemotherapy [13]. The central aim of the current retrospective single-center study was a clinicopathological characterization of an extended cohort of ssDLBCL patients, incorporating concurrent underlying indolent lymphoma histologies of all types (e.g. including marginal zone derived lymphomas).

## RESULTS

### Histopathology

The histological distribution of the concurrent indolent component was: 5 FL grade 1/2 (22.7%), 13 FL grade 3A (59.1%) and 4 marginal zone lymphoma (18.2%). Immunohistochemical data on BCL2, BCL6 and MYC expression as well as Cell of origin (COO) as determined immunohistochemically via the long-established algorithm by Hans *et al.* showing a germinal center type expression pattern (GCB-type) in 17/22 patients are briefly summarized in Table 1. Data on COO partitioned by underlying indolent lymphoma subtype are summarized in Supplementary Table 2. FISH studies were available in only 4/22 ssDLBCL patients and are summarized in Supplementary Table 3. Moreover the extent of the DLBCL component within the diagnostic sample (ranging from 30 – 90%) alongside the type of biopsy specimen as well as other complementary data derived from histopathological evaluation are summarized in Supplementary Tables 4 and 5. Cytology of malignant bone marrow infiltrates in cases of ssDLBCL upon initial diagnosis (4/22) was composed of small cells in 3/3 ssDLBCL with FL-type component whereas the case of MZL-type ssDLBCL with bone marrow involvement (1/4) exhibited large cell infiltrates.

**Table 1:** Immunohistochemical data on ssDLBCL by type of underlying indolent lymphoma histology

Antibody	FL-type ssDLBCL (n = 18)	MZL-type ssDLBCL (n = 4)
**CD10**	16/18 (88.9%)	1/4 (25%)
**MUM-1**	2/11 (18.2%)	4/4 (100%)
**BCL2**	14/16 (87.5%)	3/4 (75%)
**BCL6**	7/10 (70%)	3/3 (100%)
**MYC**	0/12 (0)	0/2 (0)
**GCB (Hans *et al.***)	16/18 (88.9%)	1/4 (25%)
**Non-GCB (Hans *et al.***)	2/18 (11.1%)	3/4 (75%)
**Ki67 (median +/- SD)**	75 (+/-17.2)	70 (+/-15)

ssDLBCL, DLBCL, diffuse large B-Cell Lymphoma; ss, simultaneous secondary; FL, follicular lymphoma; MZL, marginal zone lymphoma; GCB, germinal center B-Cell like; SD, standard deviation.

### Clinical characteristics, treatment response and outcome

**Figure 1:**
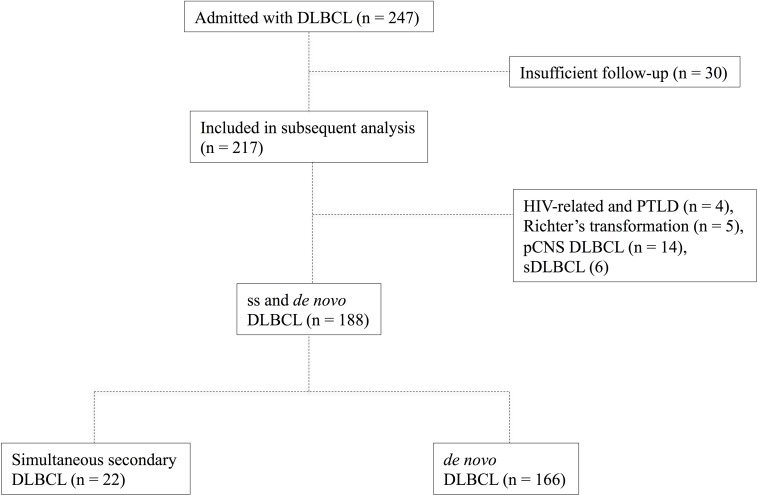
Flowchart depicting the composition of the study group and controls (*de novo* DLBCL)

**Figure 2:**
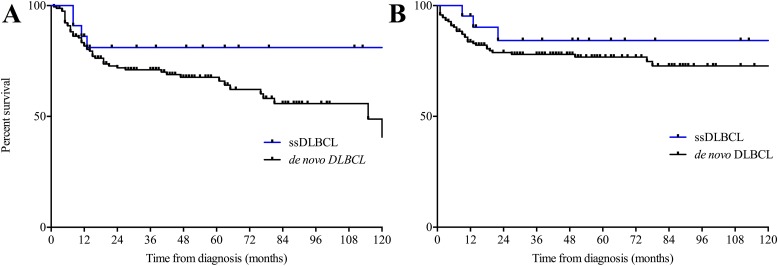
**(A)** Progression-free and overall **(B)** survival in simultaneous secondary DLBCL and *de novo* DLBCL. DLBCL, diffuse large B-Cell Lymphoma; ss, simultaneous secondary.

The mean age at diagnosis of the 22 evaluable patients was 61.3 (range 34 – 79 years). Thirteen patients (59.1%) were male. We found no significant disparities within the group of ssDLBCL based on type of concurrent indolent lymphoma. The vast majority of patients received R-CHOP induction therapy upon diagnosis (19/22) while only one of the ssDLBCL patients refused any cytoreductive treatment. In addition to six cycles of R-CHOP 6/19 patients received two additional cycles of rituximab monotherapy and another four patients received rituximab maintenance therapy over the course of two years as described [14]. Median follow-up was 43.5 months for ssDLBCL patients and 41 months in the de novo control group respectively. None of the assessed clinical parameters (Age, R-IPI, ECOG PS, LDH, extranodal sites, Ann Arbor stage) differed significantly between ssDLBCL and *de novo* DLBCL. Prognostic survey revealed further similarities as the 2-year OS observed in the current study was 84.2% and 78.0% respectively (p=0,3293; Hazard Ratio (HR) 0,6257; 95% confidence interval (CI) 0,244 to 1,61). Moreover, the 2-year PFS was 81.1% and 71.9% respectively (p=0,272; HR: 0,652; CI: 0,304 to 1,398). Clinical characteristics and therapeutic frontline approaches of all patients are summarized in Table 2 whereas composition of both study group and controls as well as survival data are depicted in Figures 1 and 2. Upon relapse a confirmatory biopsy was obtained in all cases and all but one of the ssDLBCL (4/5, 80%) exhibited high-grade histology without signs of a concurrent indolent fraction. Within the group of ssDLBCL relapse was however associated with dismal prognosis and 3/5 patients died with a median post-relapse survival of 7.5 months. Salvage treatment approaches are depicted in Supplementary Table 6. A preliminary univariate analysis followed by a Cox proportional hazard-type multivariate analysis revealed Stage III/IV (p =0.005) and R-IPI (p<0.0001) to be the only significant predictors of OS. While ECOG > 1 (p=0.001) and R-IPI (p=0.004) were the only predictors of PFS. Both multivariate analysis as well as a confirmatory propensity score matched analysis ruled out a significant impact of the presence of composite histology on OS (p=0.644 and p=0.705 respectively) and PFS (p=0.794 and p=0.236 respectively). Detailed results regarding the prognostic impact of clinical characteristics of OS and PFS are presented in Supplementary Table 7.

**Table 2:** Clinicopathological characteristics and therapeutic frontline approaches of the study group

	ssDLBCL (n = 22)	*de novo* DLBCL (n=166)	p-value
**Age (yrs.; mean + range)**	61.3 (34 – 79)	69.0 (17 – 92)	0.086
**Sex**			
female	9 (40.9%)	72 (43.4%)	
male	13 (59.1%)	94 (56.6%)	
**R-IPI**			0.3810
0	4 (18.2%)	14 (8.4%)	
1-2	10 (45.5%)	78 (47.0%)	
>2	8 (36.3%)	74 (44.6%)	
**Stage (Ann Arbor)**			0.4257
I	4 (18.2%)	27 (16.3%)	
II	7 (31.9%)	46 (27.7%)	
III	6 (27.2%)	37 (22.3%)	
IV	5 (22.7%)	56 (33.7%)	
**Extranodal sites**			0.1987
0	12 (54.6%)	70 (42.2%)	
1-2	9 (40.9%)	70 (42.2%)	
>2	1 (4.5%)	26 (15.5%)	
**ECOG PS**			0.4426
0	4 (18.2%)	34 (20.5%)	
1-2	13 (59.1%)	81 (48.8%)	
>2	5 (22.7%)	51 (30.7%)	
**LDH**			0.4701
Normal	5 (22.7%)	59 (35.5%)	
Elevated	17 (77.3%)	107 (64.5%)	
**CNS involvement at diagnosis**	0 (0%)	13 (7.8%)	
**Frontline Therapy regimen**			
R-CHOP	19 (86.4%)	130 (78.4%)	
R-Benda	0 (0%)	16 (9.6%)	
R-Tro	2 (9.1%)	7 (4.2%)	
GMALL-B-NHL 2002	0 (0%)	2 (1.2%)	
Others	1 (4.5%)	8 (4.8%)	
**Concurrent low-grade histology**			
Follicular 1-3A	18 (81.8%)	-	
MZL	4 (18.2%)	-	

DLBCL, diffuse large B-Cell Lymphoma; ss, simultaneous secondary; Yrs., years; CNS, central nervous system; LDH, Lactate dehydrogenase; ECOG; Eastern cooperative oncology group; PS, performance status; R, rituximab; CHOP, cyclophosphamide, doxorubicin, vincristine, prednisolone; Tro, Trofosfamide; Benda, Bendamustine, GMALL, German Multicenter Study Group on Adult Acute Lymphoblastic Leukemia B-NHL2002 Protocol; MZL, marginal zone lymphoma; Others, treatment refusal, other rituximab based regimen (e.g. R-Fludarabine+Cyclofosfamide) or palliative cytoreductive treatment.

**Figure 3:**
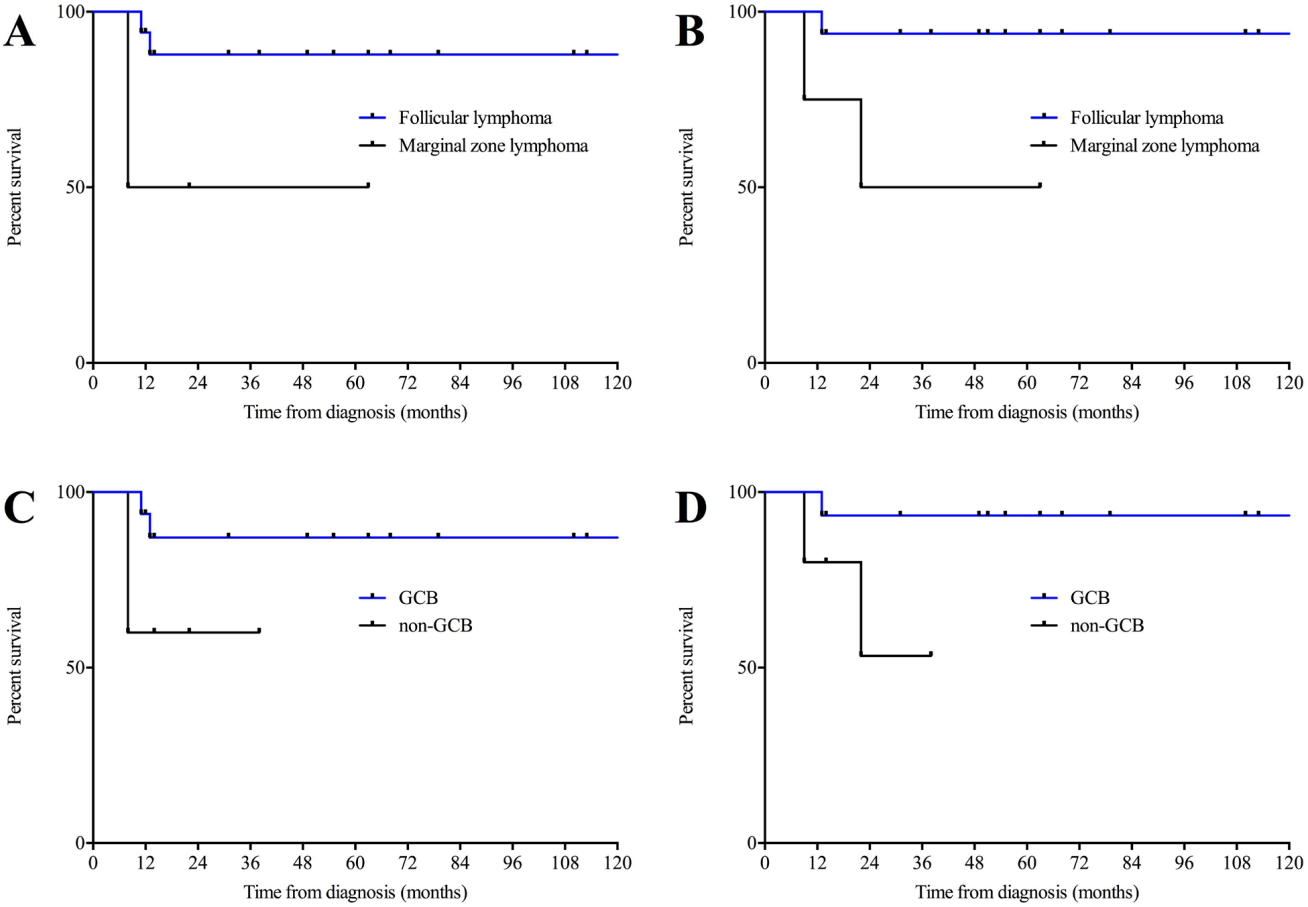
**(A)** Progression-free and overall **(B)** survival in ssDLBCL with underlying indolent lymphoma of follicular lymphoma-type and marginal zone lymphoma-type. **(C)** Progression-free and overall **(D)** survival in simultaneous secondary DLBCL of GCB and non-GCB type. GCB, germinal center B-Cell like.

In addition we detected a significant impact of underlying indolent lymphoma histology on both OS and PFS favoring follicular lymphoma-type composite disease (p=0,0299; Hazard Ratio (HR) 0,03892; 95% confidence interval (CI) 0,002 to 0,73 and p=0,0416; Hazard Ratio (HR) 0,05305; 95% confidence interval (CI) 0,0031 to 0,895 respectively; Figure 3A and 3B). Moreover, we observed a trend towards an inferior clinical outcome bordering on statistical significance for non-germinal center derived ssDLBCL according to the Hans algorithm for both OS (p=0.0579; Hazard Ratio (HR) 0,07; 95% confidence interval (CI) 0,0045 to 1,093) and PFS (p=0.1218; Hazard Ratio (HR) 0,139; 95% confidence interval (CI) 0,011 to 1,695; Figure 3C and 3D).

## DISCUSSION

In the present manuscript we outline our experience of 22 patients with ssDLBCL. Aiming to minimize selection bias 247 consecutive patients diagnosed at a single institution following centralized pathology review with a lymphoproliferative disease harboring a significant DLBCL component were recruited and evaluated retrospectively.

DLBCL is the most common subtype of NHL and implementation of R- CHOP has rendered the disease curable in about 70% of patients [15]. Even more promising outcomes, yet no sustainable cures, have been achieved in the treatment of indolent lymphomas (e.g. FL & MZL) employing immunochemotherapy [16]. The cumulative incidence of concurrent histological transformation in patients with indolent B-cell neoplasia have been incompletely evaluated. In this series depicting the clinical features and outcomes of ssDLBCL patients in the immunochemotherapy era the proportion of composite histology was 22/247 (8.9%).

All potential features of transforming or transformed indolent lymphoma were distributed similarly among ssDLBCL and *de novo* DLBCL in our current study. In keeping with the observations by Wagner-Johnston *et al.* a considerably higher percentage of patients with composite histology with transformation at initial diagnosis was treated with R-CHOP compared with the common therapeutic approaches described for indolent lymphoma [17]. The overall survival of approximately 80% at five years of follow-up was well beyond previously reported findings in transformed indolent lymphoma [6, 18]. However, Al-Tourah et al. recently discovered that patients with limited extent transformation had a significantly better survival with a 5-year post-transformation survival of 66% compared to former reports in a population based analysis [18–20].

Apart from three rituximab-era studies that identified 5/63 (7.9%) 40/878 (4.6%) and 77/2734 (2.8%) patients with tFL at the time of diagnosis, the clinical characteristics and outcomes associated with transformation at diagnosis are not well characterized [13, 17, 21]. The few studies on ssDLBCL almost exclusively included ssDLBCL of FL-type and revealed unanimously a prognosis inferior to grade III FL but similar to *de novo* DLBCL [22]. Younger mean age and sparse significant comorbidities may have contributed to the overall favorable prognosis of ssDLBCL in our current study. Another observation of note was that none of the 4/22 patients who received rituximab maintenance therapy showed signs of relapse at a median follow-up of 24.5 months. The limited size of the study group and duration of follow-up as well as the indistinct safety and therapeutic efficacy of rituximab maintenance in ssDLBCL require further prospective evaluation, especially in the light of recently demonstrated non-superiority of rituximab maintenance compared to no maintenance therapy following R-CHOP or R-CHOP-like treatment in *de novo* DLBCL [14, 23].

The most recent study by Magnano *et al.,* published during the preparation of this manuscript, was the first to characterize cases of FL/DLBCL type ssDLBCL cases with regard to Cell of origin (COO) and to explore recurrent chromosomal aberrations (*cMYC*, *BCL2* & *BCL6*) as well as *NOTCH 1/2* mutations [13]. In keeping with published studies higher grade follicular lymphoma (IIIA/B) components were represented more pronounced within the group of follicular lymphoma-type ssDLBCL (13/18 cases 72.2%) [24]. Moreover, we found our subsequent observations from FL-type ssDLBCL to be widely in keeping with results published for both *de novo* as well as ssDLBCL regarding COO whereas cases with marginal zone derived indolent lymphoma component showed a predominance of non-GCB origin [13, 25]. In addition we found a statistical trend towards a better clinical outcome among GCB-type ssDLBCL patients, which however failed to reach statistical significance in light of the small study group [13, 26]. Intriguingly, we were however able to detect an impact of underlying indolent lymphoma histology on both OS and PFS favoring follicular lymphoma-type composite disease, which reached statistical significance even within this small study group. Larger and ideally prospective trails will need to address this issue further in order to assess the necessity of treatment intensification (e.g. ASCT) in this small yet non-negligible subgroup of ssDLBCL patients.

The essential conclusion to be drawn from this study is that standard DLBCL-type immunocheotherapy (e.g. R-CHOP) induces a non-inferior response in ssDLBCL compared to *de novo* DLBCL while therapeutic intensification in the front-line setting appears expendable. To the best of our knowledge this is the first investigation to show that this may however not be the case in composite lymphoma with a non-follicular-type indolent component.

Complementarily, we found no significant rate of double-expressor or double-hit lymphoma. In the light of the newly shaped entity of high-grade B-Cell lymphoma with *MYC*, *BCL2* and or *BCL6* aberrations, which was excluded from the current study, however, this was to be expected. The impact of these observations is however diminished again by the limited size of the study group. Another point of interest for future prospective studies would be a systematical comparison of ssDLBCL with transformed indolent lymphoma with a well-documented preceding course of the underlying indolent lymphoma (“late transformations”), which are believed to arise y diverging genomic pathways [27–29]. As only few of these cases were present in our current cohort such an analysis was omitted (n = 6; data not shown).

Our observation regarding the predominance of high-grade morphology without composite indolent fractions upon relapse may partially be attributed to the limited time of follow-up as recent studies revealed a prolonged progression-free survival in both transformed and non-transformed FL patients in the immunochemotherapy era and the vast majority of ssDLBCL patients in our study received R-CHOP induction treatment [6]. As indolent lymphoma such as FL have to be considered incurable even in the immunochemotherapy era a subsequent accumulation of relapse derived from the indolent fraction of the disease still is to be expected in our cohort over time although recent studies suggested a reduced rate of such adverse events in the era of immunochemotherapy [18]. Intensive long-term follow-up for these patients is therefore clearly warranted.

Limitations of the present study primarily include its limited sample size, especially relativizing our sub group analysis among ssDLBCL cases based upon histology of the underlying lymphoma. The statistical significances we derived from our data need to be validated in larger and if possible prospective trails before definitive suggestions regarding personalized risk-assessment and ultimately treatment intensifications should be made. Other deficits are principally those typically associated with retrospective studies. Baseline clinical characteristics are derived from the information in the medical records, which harbors a potential for fragmentary data, especially while the vast majority of deaths within the study group were lymphoma related, the fraction of patients lost to follow-up due to non-lymphoma related death can not be securely estimated from our data. Another shortcoming inherent to the retrospective design of the study is the potential of a selection bias of indistinct extent. This aspect is of particular interest in such a limited study group and the current data need therefore to be interpreted as preliminary.

## CONCLUSION

In summary, our findings underline the close clinicopathological relationship between ssDLBCL and *de novo* DLBCL advocating intensive R-CHOP-like treatment upon initial diagnosis. Moreover both the short- as well as the intermediate-term course of the disease is predominantly determined by the high-grade component with a trend towards a more favorable outcome among germinal center derived ssDLBCL and those with a concurrent follicular lymphoma-type component. A non-FL-type composite DLBCL may however require treatment intensification.

## MATERIALS AND METHODS

### Patients

To evaluate clinical and prognostic characteristics, we reviewed our databases to identify patients with ssDLBCL admitted to our institution (University Hospital Schleswig Holstein Campus Luebeck - covering the population in the area of southern Schleswig Holstein consisting of approximately 1.000. 000 inhabitants) between January 2010 and July 2017, excluding patients with insufficient follow-up (30 patients referred to other centers following primary diagnosis and subsequent loss of follow-up), primary central nervous system (pCNS) DLBCL (n = 14), PTLD and HIV-related Lymphoma (n = 4), Richter's transformation as well as transformed indolent lymphoma (n = 5 & 6 respectively). Clinical information was collected from the original files, and data concerning performance status (Eastern Cooperative Oncology Group [ECOG]), disease extent, treatment modalities, response rate, relapse pattern, cause of death and survival were recorded, as were initial serum levels of lactate dehydrogenase (LDH). Staging was performed according to the Cotswold modifications of the Ann Arbor classification [30]. Moreover all patients were assessed according to the revised international prognostic index (R-IPI) [31].

### Centralized histological assessment and criteria for ssDLBCL

247 consecutive patients with DLBCL (double-hit lymphomas were excluded) diagnosed in accordance with the latest edition of the WHO classification of tumors of the lymphoid system (Reference center for haematopathology, University Hospital of Schleswig-Holstein, Campus Luebeck) treated at our institution were identified over the course of the above mentioned time period [3].

The histological criteria for ssDLBCL were defined as the presence of a variable DLBCL component in the lymph node biopsy (both open lymph node excision biopsy as well as needle core biopsy samples were included) otherwise showing FL or other indolent lymphoma infiltrates. By WHO recommendations, a composite histology component was defined as sheets of large cells infiltrating the underlying indolent lymphoma and lacking follicular architecture defined by the absence of dendritic cells in cases of underlying FL (CD23). In cases of underlying marginal-zone-derived indolent lymphoma the populations of small and large B-cells were more diffusely mixed and therefore the diagnosis of ssDLBCL was only established in cases where a concurrent yet sufficiently definable indolent lymphoma component could be proven both morphologically as well as immunophenotypically as described [32]. The percentage of the DLBCL component within the diagnostic biopsy was quantified and cell of origin (COO) was assessed employing the immunohistochemical algorithm described by Hans *el al.* [33]. Histological slides were reviewed by HM and NG in order to confirm diagnosis. Antibodies used are summarized in Supplementary Table 1.

Fluorescence-in-situ-hybridization (FISH) for chromosomal aberrations affecting *BCL2* / *BCL6* / *cMYC* was not routinely performed but reserved for cases with suspicious morphology and high proliferative activity (Ki67 > 90%).

### Treatment, response assessment, staging and outcome

Following base line staging investigations according to standard procedures patients were treated with an (immuno-) chemotherapy regimen of the treating physician's choice with R-CHOP-like immunochemotherapy serving as the institutional standard [34]. Bone marrow aspirates (+FACS) and trephine biopsies were routinely performed at initial diagnosis and thereafter in case of prior involvement or clinical suspicion of bone marrow infiltration.

Treatment response was evaluated in accordance with widely established criteria and definitions of complete (CR) and partial response (PR) as well as the method for calculation of overall (OS) and progression free survival (PFS) were employed according to standard definitions [34]. FDG-PET-Scans were not employed on a routine basis for all patients.

### Ethics statement

Written informed consent for retrospective analysis of their clinical data and scientific use of biopsy material was obtained from all patients and the study was approved by the ethics committee of the University of Luebeck (reference-no 17-266).

### Statistics

Time to progression and overall survival (PFS, OS) were calculated from the date of initial diagnosis. Survival (PFS and OS) was primarily estimated by means of the Kaplan–Meier method and the univariate log-rank test. Characteristics found to be associated with either OS or PFS with at least a trend towards statistical significance (p < 0.07) were included in a subsequent multivariate proportional hazard model (Cox proportional hazard) alongside composite histology. Given the non-matched composition of the ssDLBCL and *de novo* DLBCL groups we conducted a confirmatory propensity score matching approach (matching for age, sex, ECOG, LDH, Stage, R-IPI) resulting in two matched groups (ssDLBCL and *de novo* DLBCL; n = 22 each) followed again by a univariate log-rank test for difference in PFS and OS with regard to the presence of composite histology. Differences among patient subgroups were assessed by using the chi- square as well as the Mann-Whitney-U-test was used when appropriate. All statistical investigations were conducted using GraphPad PRISM 6 and SPSS 24 (IBM).

## SUPPLEMENTARY MATERIALS FIGURES AND TABLES


