# Supplementary material for: Indolent lymphoma with composite histology and simultaneous transformation at initial diagnosis exhibit clinical features similar to de novo diffuse large B-cell lymphoma
Source: Oncotarget. 2018 Apr 13;9(28):19613–22. doi: 10.18632/oncotarget.24701 (PMC5929412; doi:10.18632/oncotarget.24701)
Supplement: Supplementary file 1 [file oncotarget-09-19613-s001.pdf]

Indolent lymphoma with composite histology and simultaneous transformation at initial diagnosis exhibit clinical features similar to *de novo* diffuse large B-Cell lymphoma

SUPPLEMENTARY MATERIALS

Supplementary Table 1: Antibodies used

Antibody	Supplier	Clone
Bcl2	Lab Vision	100/D5
Bcl6	Dako	BG-B6p
CD10	Menarini	56C6
MUM-1 (Irf4)	Dako	Mum 1P
Ki-67	Dako	Mib-1
MYC	Roche	Y69

Supplementary Table 2: Data on COO partitioned by underlying indolent lymphoma subtype

Indolent component	Cell of origin		
	GCB	Non-GCB	Total
Follicular lymphoma grade I / II	4	1	5
Follicular lymphoma grade IIIa/b	12	1	13
Marginal zone lymphoma	1	3	4

Supplementary Table 3: FISH studies in ssDLBCL patients

Probe	Positive	Negative
BCL2	0/4	4/4
BCL6	0/3	3/3
cMYC	0/3	3/3

Positive and negative controls were routinely used as generally recommended securing the validity of the assays.

Supplementary Table 4: Extent of the DLBCL Component, concurrent indolent histology, COO and type of biopsy specimen for all ssDLBCL cases

Patient ID	DLBCL component (%)	Concurrent indolent lymphoma histology	COO (Hans *et al.*)	Type of biopsy specimen
ssDLBCL-1	60	FL Grade I/II	GCB	EB
ssDLBCL-2	90	FL Grade I/II	GCB	NCB
ssDLBCL-3	90	FL Grade I/II	GCB	EB
ssDLBCL-4	90	FL Grade I/II	non-GCB	NCB
ssDLBCL-5	50	FL Grade II	GCB	NCB
ssDLBCL-6	50	FL Grade IIIA	GCB	EB
ssDLBCL-7	50	FL Grade IIIA	GCB	EB
ssDLBCL-8	70	FL Grade IIIA	GCB	EB
ssDLBCL-9	80	FL Grade IIIA	GCB	NCB
ssDLBCL-10	80	FL Grade IIIA	GCB	EB
ssDLBCL-11	70	FL Grade IIIA	GCB	NCB
ssDLBCL-12	80	FL Grade IIIA	GCB	NCB
ssDLBCL-13	70	FL Grade IIIA	GCB	NCB
ssDLBCL-14	60	FL Grade IIIA	GCB	EB
ssDLBCL-15	80	FL Grade IIIA	non-GCB	EB
ssDLBCL-16	70	FL Grade IIIA	GCB	EB
ssDLBCL-17	40	FL Grade IIIA	GCB	EB
ssDLBCL-18	30	FL Grade IIIA	GCB	EB
ssDLBCL-19	80	MALT lymphoma	GCB	NCB
ssDLBCL-20	75	MALT lymphoma	non-GCB	NCB
ssDLBCL-21	50	MALT lymphoma	non-GCB	EB
ssDLBCL-22	90	MALT lymphoma	non-GCB	EB

COO, Cell-of-origin; FL, follicular lymphoma; MALT, mucosa-associated lymphoid tissue; GCB, germinal center type B-Cell; NCB, needle core biopsy; EB, excision biopsy.

Supplementary Table 5: Baseline characteristics according to underlying indolent lymphoma component partitioned by follicular lymphoma grade I/II, follicular lymphoma grade III, marginal zone lymphoma

**Patient Characteristics**			
	**Follicular lymphoma grade I/II (n = 5)**	**Follicular lymphoma grade III (n = 13)**	**Marginal zone lymphoma (n = 4)**
**Age (yrs.; mean + range)**	65 (34 – 78)	58.5 (41 – 79)	65.8 (52 – 77)
**Sex**			
female	3 (60%)	4 (31%)	2 (50%)
male	2 (40%)	9 (69%)	2 (50%)
**R-IPI**			
0	0 (0)	4 (31%)	0 (0)
1-2	1 (20%)	7 (54%)	2 (50%)
>2	4 (80%)	2 (15%)	2 (50%)
**Stage (Ann Arbor)**			
I	0 (0)	2 (15%)	2 (50%)
II	1 (20%)	6 (46%)	0 (0)
III	2 (40%)	4 (31%)	0 (0)
IV	2 (40%)	1 (8%)	2 (50%)
**Extranodal sites**			
0	2 (40%)	9 (69%)	1 (25%)
1-2	3 (60%)	4 (31%)	2 (50%)
>2	0 (0)	0 (0)	1 (25%)
**ECOG PS**			
0	1 (20%)	3 (23%)	0 (0)
1-2	2 (40%)	9 (69%)	2 (50%)
>2	2 (40%)	1 (8%)	2 (50%)
**LDH**			
Normal	1 (20%)	3 (23%)	1 (25%)
Elevated	4 (80%)	10 (77%)	3 (75%)
**CNS involvement at diagnosis**	0 (0)	0 (0)	0 (0)
**Frontline Therapy regimen**			
R-CHOP	4 (80%)	11 (84%)	4 (100%)
R-FC	0 (0)	1 (8%)	0 (0)
R-Tro	1 (20%)	1 (8%)	0 (0)

Yrs., years; CNS, central nervous system; LDH, Lactate dehydrogenase; ECOG; Eastern cooperative oncology group; PS, performance status; R, rituximab; CHOP, cyclophosphamide, doxorubicin, vincristine, prednisolone; FC, Fludarabine, Cyclofosfamide; Tro, Trofosfamide.

Supplementary Table 6: Salvage therapies in ssDLBCL patients upon relapse

Patient ID	1st line	2nd line	3rd line	status
ssDLBCL-20	R-CHOP 21	R-ICE	R-Benda	Dfd
ssDLBCL-22	R-CHOP 21+ 2 R-Mono	R-DHAP	R-Tro	Dfd
ssDLBCL-2	R-Tro	n	n	Dfd
ssDLBCL-16	R-Tro	R-Tro	n	Alive at 51 months follow-up
ssDLBCL-5	R-CHOP 21+ 2 R-Mono	R-DHAP + HDBEAM + ASCT	n	Alive at 186 months follow-up

Tro, Trofosfamide; DHAP, Dexamethason + high dose Cytarabin + Cisplatin; ICE, Ifosfamide, Carboplatin, Etoposide; HDBEAM, high dose carmustine, etoposide, cytarabine and melphalan; ASCT, autologous stem cell transplantation; Dfd, dead from disease; n, no treatment.

Supplementary Table 7: Univariate (log rank test) and multivariate (Cox proportional hazard) analysis for Overall (OS) and Progression-free survival (PFS) in the study group (p-values)

Characteristics	Univariate OS	Univariate PFS	Multivariate OS	Multivariate PFS
**Age > 60**	0.093	0.274	-	-
**Sex**	0.756	0.692	-	-
**ECOG > 1**	**< 0.0001**	**< 0.0001**	0.052	**0.001**
**LDH**	0.09	**0.033**	-	0.941
**Stage III/IV**	0.067	**0.002**	**0.005**	0.310
**R-IPI**	**< 0.0001**	**< 0.0001**	**< 0.0001**	**0.004**
**Composite histology**	**0.302**	**0.272**	**0.644**	**0.794**

Characteristics found to be associated with either OS or PFS with at least a trend towards statistical significance ($p < 0.07$) were included in the multivariate proportional hazard model alongside composite histology.

ECOG; Eastern cooperative oncology group; LDH, Lactate dehydrogenase; R-IPI, Revised international prognostic index; ssDLBCL, simultaneous secondary diffuse large B-cell lymphoma.
